# Change of Air. Pulo Pinang

**Published:** 1831

**Authors:** 


### XXVII.

### CHANGE OF AIR. PULO PINANG.

THE following remarkable instance of the salutary effects of CHANGE OF AIR, more especially of a change from the plain to the mountain, is taken from a work published in India by Dr. Ward, but not circulated or known in this country. The work will be noticed more at large in other parts of this Journal.

"An officer of rank, 32 years in India, had been for the last 14 years of his life, subject to repeated attacks of disease, originating in a morbid affection of the colon, a little above the caput cæcum, at one time producing distressing dysenteric symptoms, at another obstinate alvine obstruction. He had been subjected to various courses of medicine, and various methods of treatment, each only with temporary benefit. Five months after his arrival on this Island, he had a recurrence of the dysentery, which was relieved by a course of blue pill, Ipecacuan and Opium, with occasional doses of oil. He afterwards ascended the Great Hill, remained there two months, and returned comparatively well. Six months after, the disease again recurred, and then was evidently accompanied with deranged liver. These were palliated by a course of mercurials, castor oil and emollient enemata, with occasional doses of hyosciamus.—On the evening of the 31st of January, 1830, he had a recurrence of the violent spasmodic pain in the region of the colon, he passed a wretched night, had repeated chills and flushes; felt great depression of spirits and total failure of strength. He had recourse to the measures above stated, with only slight benefit. On the 7th February, the following symptoms were noted. He was much emaciated; his cheeks sunk; his face and eyes sallow, and patches of yellow appeared about the lips and here and there upon the face. His eyes were dull; his forehead was warm, and the skin generally warmer than natural; his voice was faint and low, and conversation was difficult; pulse 120, irritable, compressible; tongue coated with an orange fur. His dejections were bilious, copious, offensive and mucous. Pain in the region of the colon, relieved by rubbing and hot bottles. A distressing dry cough came on four days ago; fulness and pain were present in the right hypochondriac region, and during the cough, he felt as if something was tearing the epigastrium. He had repeated flushings of the face, and occasional chills; a sense also of fluctuation as if water trickled down his back, and a cold clammy perspiration about the loins. His strength was exhausted; appetite gone, and there was increased thirst.—In consultation it was determined that the case was one of great emergency, that the symptoms threatened the occurrence of abscess of the liver superadded to the disease of the colon; and that the object of remedial measures was to support the system, until it could be acted upon by mercurials. Change of air, and that immediately, suggested itself, as the most likely means of effecting this; and as there was a difference at that time of 14° between the valley where he was living and the Great Hill, he was recommended to remove to the latter without the least delay. He accordingly ascended the mountain in a Ton-jon, (a sort of chair borne on mens' shoulders) and reached the summit about 6½ A. M. refreshed at every step by the bracing breeze, and not in the least fatigued by the removal. The hill air acted upon him like a charm. On the very first day, all the symptoms were relieved, his spirits gradually improved, and by means of a gentle course of mercurial medicines, until the mouth became slightly affected, but not to salivation, with a seton in the side, and mild nutrient diet, his general health was greatly restored; his strength had much increased, and the state of his stomach and bowels was also improved, tho' he was still subject to occasional attacks of spasm and obstruction of the colon. On the 1st of June, he was almost as completely re-established in health as he could have been by a trip to England, tho' of course the improvement cannot be expected to be permanent; and return to a hot climate will most likely be followed by a relapse.

I am inclined to attribute the successful issue of this case, entirely to the beneficial operation of the mountain air, as had he continued in the Valley, where the heat was then oppressively great, the thermometer being at 88° in the middle of the day, I have no doubt that he would have sunk from exhaustion."

The salutary effects of CHANGE OF AIR are supposed to be well known—but they are very imperfectly known, comparatively speaking. If, indeed, we reflect, for a moment, on the difference that must necessarily exist between the air of a city or a valley, and that of a mountain or open country, we must be convinced of the importance of this difference in respect to health. It is not only probable but certain, that all or most of the deleterious agents emanating from the earth on which we live, hover in the lower strata of air, and consequently become concentrated in the atmosphere of cities and valleys. As we ascend the mountains, we get beyond the reach of these agents; and on the open plains they are greatly diluted, if not dissipated, by the breezes which sweep uninterruptedly along the surface of the soil. But independently of these considerations, there is something so exhilarating and salutary in a constant change of the fluid by which "we live and move and have our being," that it is wonderful the wealthy citizens of these islands do not more frequently avail themselves of such a powerful preservative of life and health, than they now do! It is rather unfortunate that the mountains of Wales and Scotland are so far removed from Modern Babylon; but still the distance is trifling, when compared with the stock of health which a Summer or Autumnal tour confers on those who breathe the pure air of these places. We could illustrate this subject by many striking examples; but must defer the illustration for the present.

---

